# A draft genome of Escherichia coli sequence type 127 strain 2009-46

**DOI:** 10.1186/1757-4749-6-32

**Published:** 2014-09-01

**Authors:** Aaron E Darling, Jessica McKinnon, Paul Worden, Jerran Santos, Ian G Charles, Piklu Roy Chowdhury, Steven P Djordjevic

**Affiliations:** 1 ithree Institute, University of Technology Sydney, Broadway Street, 2007 Ultimo, Australia; 2 NSW Department of Primary Industries, Elizabeth Macarthur Agricultural Institute, Woodbridge Road, 2568 Menangle, Australia

**Keywords:** Escherichia coli 2009-46, Genome, Sequencing

## Abstract

**Background:**

*Escherichia coli* are a frequent cause of urinary tract infections (UTI) and are thought to have a foodborne origin. *E. coli* with sequence type 127 (ST127) are emerging pathogens increasingly implicated as a cause of urinary tract infections (UTI) globally. A ST127 isolate (2009-46) resistant to ampicillin and trimethoprim was recovered from the urine of a 56 year old patient with a UTI from a hospital in Sydney, Australia and was characterised here.

**Results:**

We sequenced the genome of *Escherichia coli* 2009-46 using the Illumina Nextera XT and MiSeq technologies. Assembly of the sequence data reconstructed a 5.14 Mbp genome in 89 scaffolds with an N50 of 161 kbp. The genome has extensive similarity to other sequenced uropathogenic *E. coli* genomes, but also has several genes that are potentially related to virulence and pathogenicity that are not present in the reference *E. coli* strain.

**Conclusion:**

*E. coli* 2009-46 is a multiple antibiotic resistant, phylogroup B2 isolate recovered from a patient with a UTI. This is the first description of a drug resistant *E. coli* ST127 in Australia.

## Background

*Escherichia coli* infections of the urinary tract are among the most frequent infections reported in the developed world with an estimated 130-175 million cases per annum worldwide [1]. *E. coli* that cause urinary tract infections (UTI) are classified as uropathogenic *Escherichia coli* (UPEC), a subgroup of extraintestinal pathogenic *E. coli* (ExPEC). ExPEC also cause a range of afflictions including meningitis, septicaemia, and pneumonia and are genotypically and phenotypically distinct from diarrhoegenic *E. coli* (DEC) [2]. ExPEC are thought to be acquired orally via the consumption of contaminated food and are considered to be zoonotic pathogens [3-5]. The emergence of multiple antibiotic resistance among ExPEC poses a serious health threat; antibiotics are an important treatment strategy for controlling UTI.

Multilocus sequence typing (MLST) is currently the gold standard for characterising *E. coli* causing UTI. No clear diagnostic markers are available for identifying *E. coli* causing UTI, but several sequence types (ST) including ST131, ST405, ST95, ST65, ST127, and ST10 are recognised UTI pathogens [6]. ExPEC ST127 are described as community-acquired and highly virulent zoonotic pathogens [3,6] but to our knowledge there are no genome sequences representing antibiotic resistant isolates of this emerging pathogen. Studies of *E. coli* causing UTI in Australia have focussed on characterising ST131 [7,8] and serogroup O75 isolates belonging to clonal complex 14 [9].

Here we describe the genome sequence of *E. coli* ST127 isolate 2009-46, a mid-stream urinary tract isolate from a 56 year old patient from the Sydney Adventist Hospital (SAN clinic) resistant to ampicillin and trimethoprim.

## Methods

The isolate was supplied on a Sensi-agar plate from the SAN laboratories in Sydney, Australia. To confirm pure culture, a loopful of the isolate was streaked onto a Luria Bertani (LB) Agar plate and incubated at 37°C for long term storage in minus 80°C as a glycerol stock. A single colony was picked from the plate and subcultured in 10 mL LB broth at 37°C overnight. To prepare the glycerol stock culture 7 mL of the overnight was used, and genomic DNA was prepared from the remaining 3 mL. Genomic DNA for sequencing was prepared using the ISOLATE II gDNA extraction kit from Bioline.

### Genome sequencing

DNA was quantified using qubit flourimetry and 0.5 ng of gDNA was used as template to construct the sequencing library, using the Illumina Nextera XT library preparation protocol following the manufacturer’s instructions. However, the "PCR Clean-Up" and "Library Normalization" steps were omitted and size selection was instead performed by running balanced and pooled samples in a 1% agarose gel and excising the 600 bp to 1200 bp region of interest. The DNA was then purified from the agarose using Promega’s Wizard SV Gel and PCR Clean-Up System. Finally, an Agilent 2100 Bioanalyzer, with a High Sensitivity DNA Kit, was used to quantitate the pooled DNA library before loading onto the MiSeq with other multiplexed samples. Two MiSeq runs were carried out, one with paired-end 250 nt reads on MiSeq V2 chemistry and another with paired-end 300 nt reads on V3 chemistry. The first library was found to have an average insert size of 368 +/- 157 nt, while the second library had inserts with an average size 497 +/- 118 nt.

### Assembly and annotation

The genome was assembled using the A5-miseq pipeline, a version of the A5 pipeline [10] that has been revised to process reads up to 500 nt long. Briefly, the A5-miseq pipeline consists of five stages: (1) read quality filtering and error correction, (2) contig assembly, (3) permissive draft scaffolding, (4) misassembly detection, and (5) conservative scaffolding. The revised A5 pipeline uses a new version of idba_ud that uses read pairing information, and that has been modified to accept reads up to 500 nt long and to construct *de Bruijn* graphs with *k*-mers up to 500 nt. These modifications provide substantial improvements in assembly contiguity.

The genome was annotated with the RAST annotation system using FigFAM release 70 [11]. Putative antibiotic resistance genes and other genes of interest identified by RAST annotation were manually curated using the NCBI ORF finder and iterative BLASTn and BLASTp searches.

## Quality assurance

The A5 pipeline includes a quality checking step that detects putative misassemblies by identifying clusters of read pairs that map to disjoint locations in the assembled genome. This method did not detect any putative misassemblies.

## Initial findings

**Figure 1:**
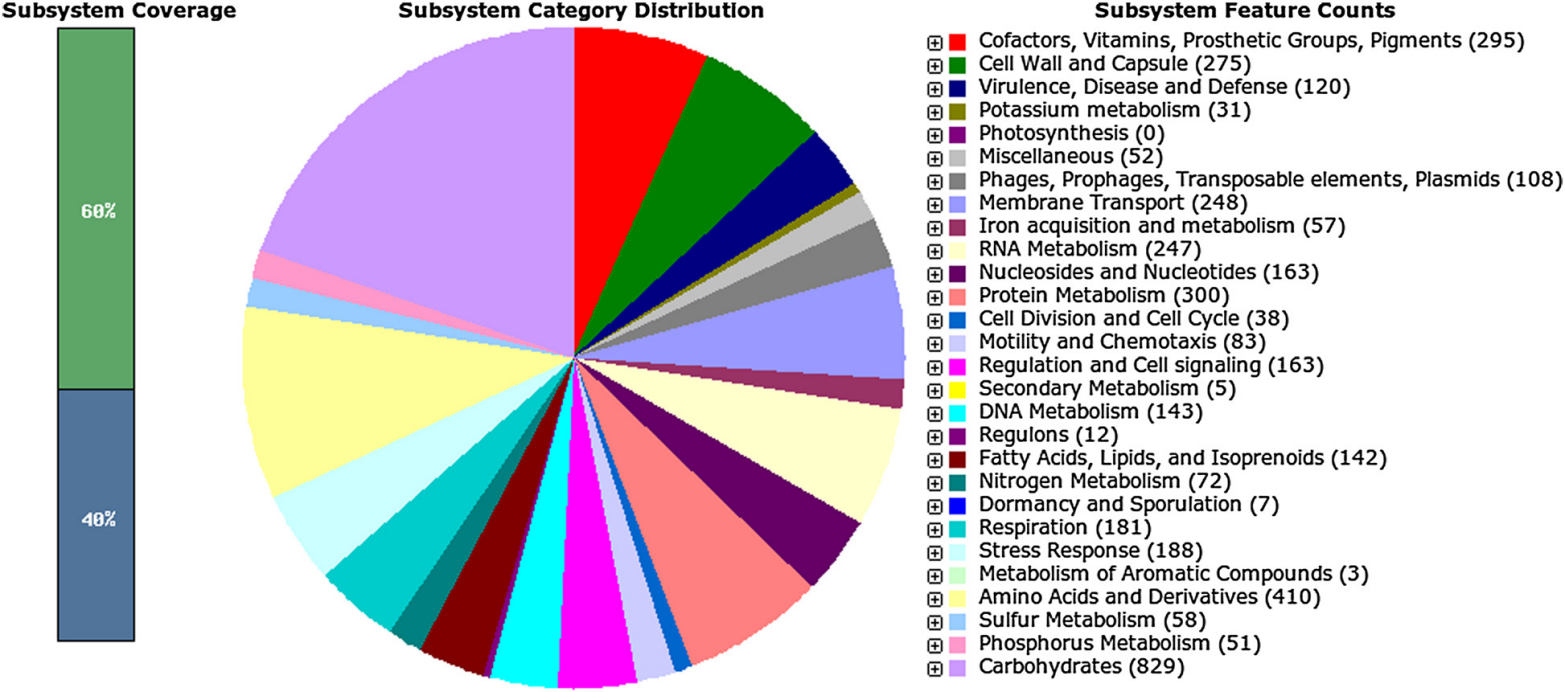
**Subsystems in** ***E. coli* 2009-46.**

**Figure 2:**
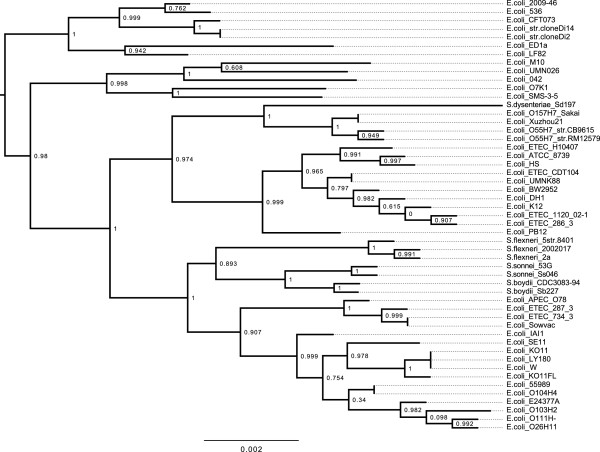
**Phylogeny of *E. coli * and *Shigella * including the 2009-46 isolate.** A phylogeny inferred on a concatenated set of codon alignments from 37 universally conserved genes is shown, as calculated by PhyloSift [12] and FastTree2 [13]. The phylogeny has been rooted on the branch leading to *Salmonella* and internal nodes are labeled with SH-like support values.

Sequencing generated 1,702,236 read pairs for a total of 483,658,987 nt that were assembled to reconstruct the 5,139,229 bp genome of *E. coli* 2009-46 in 89 scaffolds, with a scaffold N50 of 161 kbp and an N90 of 30.8 kbp. The raw (unfiltered) coverage is 94x, and after read filtering the assembly has a median depth of coverage of 61x. The annotation of this assembly identified 5084 predicted CDS and 106 predicted RNA genes. 19 genes were identified as possibly missing from the assembly by the RAST system. The overall functional profile of the genome is shown in Figure 1. We conducted a phylogenetic analysis of *E. coli* 2009-46 using the PhyloSift software [12] to identify the most closely related organism with an available reference genome. PhyloSift works by identifying homologs in the draft genome to a set of 37 genes that are universally conserved among bacteria and archaea and present in single copy. It then adds any homologs found in the draft genome to an existing multiple sequence alignment containing the 37 genes from a subset of all genomes publicly available in the NCBI and EBI databases that is chosen to span the phylogenetic diversity of these databases. The PhyloSift reference database includes only a single representative from groups of closely related organisms. To gain additional resolution in the *Escherichia*, we used PhyloSift to construct a multiple alignment of the 37 marker genes from all finished *E. coli* genomes available in the NCBI database as of September 2013. We then inferred a phylogeny from that alignment using FastTree2 [13]. The resulting analysis, shown in Figure 2, identified *E. coli* 536 as the most closely related isolate with a finished genome available, although there was some uncertainty in the 37 gene alignment as to whether *E. coli* 2009-46 diverged on the same lineage as *E. coli* 536. We used the closely related genome of *E. coli* 536 as a reference for further comparative analysis.

**Figure 3:**
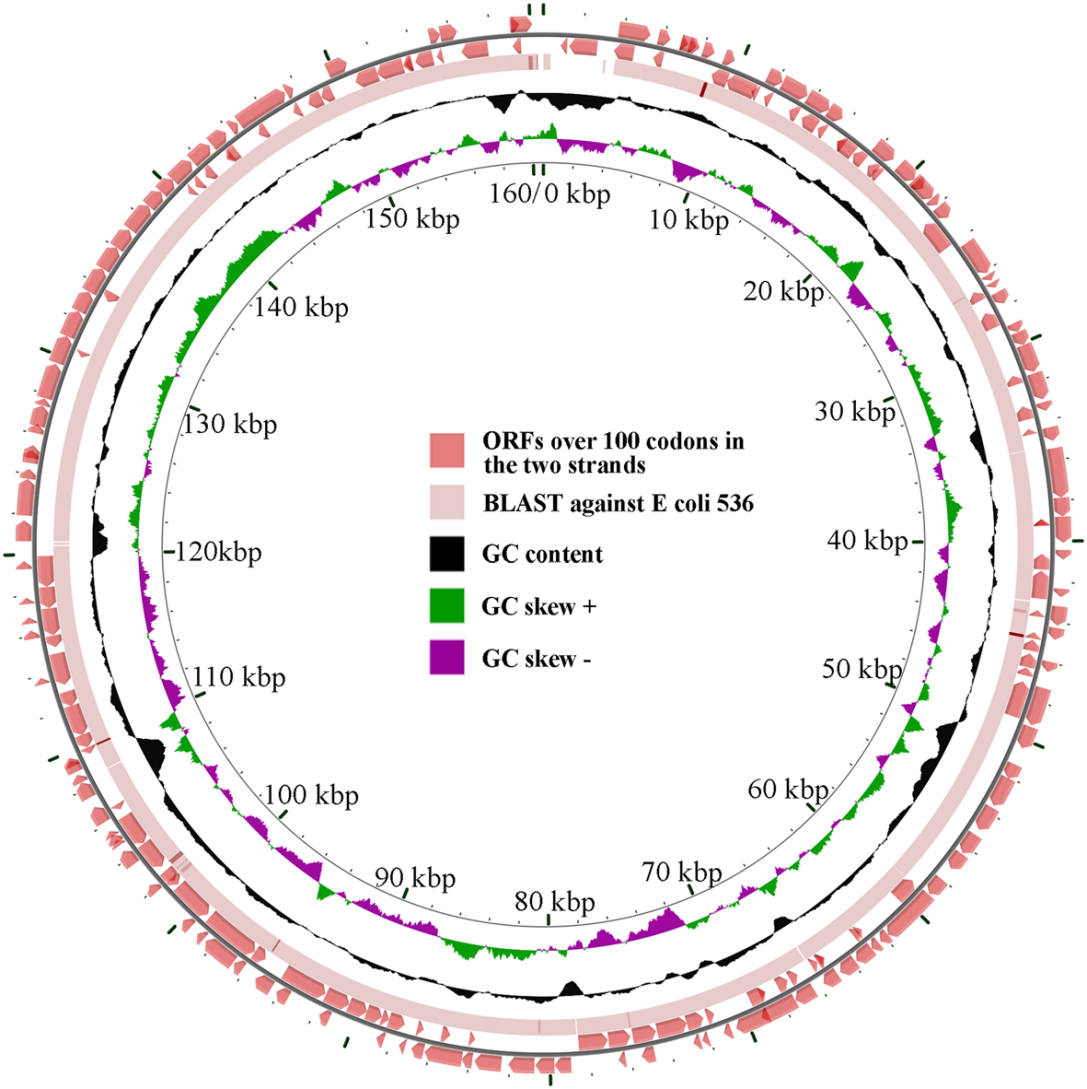
**CGview plot of the *E. coli * 2009-46 genome.** The two outermost circles in the figure contain a series of arrows in opposite directions representing predicted ORFs (greater than 100 codons) on the two strands of DNA sequence. The solid line, forming the third ring (from outside) indicates BLASTn analysis (set with a cutoff of 1e-10) of the isolate against the *E. coli* 536 genome. Relative GC content along the length of the genome is plotted as a graph in the black circle. The GC content clearly indicates multiple regions of GC content variation along the genome, possibly indicating lateral gene transfer events.

The scaffolds of *E. coli* 2009-46 were reordered to match the order in the finished genome of the closely related strain *E. coli* 536 using the Mauve Contig Mover [14]. After reordering, the genomes had 82 predicted rearrangement breakpoints. Many of these cluster in regions containing annotated transposase genes and multi-copy transporter gene families, suggesting either homology-mediated rearrangement or misassembly has occurred at these repetitive sequences. To further characterize the structure of the genome we used the CGview webserver [15] to plot matches to annotated proteins and the GC skew of the genome, with scaffolds ordered according to the *E. coli* 536 reference. The CGview plot is shown in Figure 3. Of note, the GC skew in *E. coli* 2009-46 genome appears to fluctuate frequently. This pattern is in sharp contrast to the GC skew of the *E. coli* 536 reference, which shows a strong pattern coinciding with the chromosome’s replication arms (data not shown). This suggests that either *E. coli* 2009-46 has undergone substantial genome rearrangement in the recent past, that the true genome arrangement may not match the *E. coli* 536 reference very closely, that undetectable misassembly errors exist in the *E. coli* 2009-46 genome, or that some combination of these three situations exists. We note that our assembly pipeline contains a step to detect and fix misassembly errors; none were found in the genome of *E. coli* 2009-46.

Comparison of the gene content between *E. coli* 2009-46 and the finished *E. coli* 536 reference genome identified 164 annotated gene functions predicted to be present only in *E. coli* 2009-46. Included among these are several genes related to scavenging iron, a type VII secretion system, an IncF conjugation system, mediators of hyperadherence, and copper and mercury resistance genes. The full list of gene functions found only in 2009-46 and those which 2009-46 lacks relative to the reference isolate are listed in Additional files 1 and 2, respectively.

The *bla*_TEM1_ gene, conferring resistance to ampicillin, was present on scaffold 78.1 (2551 nt), while the *sul2-strA-strB* genes conferring resistance to sulphonamides and streptomycin was located on scaffold 67.1, which was 5064 nt long. Ends of both the scaffolds had a partial copy the insertion element IS*26*. The isolate also houses a clinical class 1 integron and two associated resistance genes on scaffold 71.1. One of the two resistance genes is a variant of dihydrofolate reductase (*dhfr*) gene which provides trimethoprim resistance to isolates and the other confers resistance to aminoglocoside antibiotics (*aadA*). However the scaffold, 71.1, is 3,863 nt long and also has a copy of IS*26* at both ends. We identified the presence of the 3’-CS of a class 1 integron on scaffold 58.1 (6679 nt long), that had an IS*26* on one end and an IS*1* element on the other. Presence of IS*26* elements at both ends of seven scaffolds has resulted in scaffold breaks around a region of the genome, which most likely harbours a complex resistance locus (CRL), during the assembly of the genome sequence. We were therefore unable to confirm the exact genomic location of the CRL or resistance genes.

## Antibiotic resistance profile

The antibiotic resistance profile of *E. coli* 2009-46 was experimentally determined using the disk diffusion method. This strain was found to be resistant to Ampicillin, Trimethoprim, Sulphafurazole, Tetracycline, Streptomycin, Apramycin, Kanamycin, and Azithromycin. A full list of antibiotics tested and *E. coli* 2009-46 susceptibility is provided in Additional file 3.

To better understand the genomic basis for the observed antibiotic resistance traits, the genome was searched for specific genes known to confer antibiotic resistance. A listing of these genes and their presence or absence in *E. coli* 2009-46 is provided in Additional file 3.

## Future directions

Improved efficiency of clinical genomics pipelines will eventually enable fine-scale epidemiological monitoring of *E. coli* outbreaks in real time. When fully developed, this capacity will influence clinical and public health decisions related to treatment and control of pathogen outbreaks. Genomic data such as is presented here will aid in the interpretation of data from future outbreaks.

## Availability of supporting data

The draft genome assembly has been submitted to NCBI and is associated with BioSample accession SAMN02725027. Genome annotations are available from the RAST web server under accession 562.3620. The Illumina sequence reads have been deposited to the Short Read Archive under accessions SRX514806 and SRX514807. CDS: Coding DNA sequences; ORF: Open Reading frame; RAST: Rapid annotation using subsystem technology; A5: Andrew and Aaron’s Awesome Assembly; gDNA: genomic DNA; nt: Nucleotides;

## Competing interests

The authors declare that they have no competing interests.

## Authors’ contributions

JM extracted DNA and conducted all laboratory based analysis on the isolate. PW and JS constructed Illumina libraries and sequenced them. AED and PRC conducted the assembly, analysis, and data deposition. AED, SD, JM and PRC wrote the paper. IGC and PRC provided general direction. All authors read and approved the final manuscript.

## Supplementary Material

Additional file 1 Gene functions (as identified by RAST subsystems) found to be present in the newly sequenced E. coli 2009-46 isolate but not the E. coli 546 reference genome. Click here for file

Additional file 2 Gene functions (as identified by RAST subsystems) found to be present only in the E. coli 546 reference genome in a pairwise comparison with E. coli 2009-46. Click here for file

Additional file 3 Details on PCR cartography, virulence gene searches, and antibiotic resistance assays. Click here for file

## References

[B1] Russo TA Johnson JR Medical and economic impact of extraintestinal infections due to *Escherichia coli*: focus on an increasingly important endemic problem Microbes Infect 2003 5 5 449 456 10.1016/S1286-4579(03)00049-2 12738001

[B2] Russo TA Johnson JR Proposal for a new inclusive designation for extraintestinal pathogenic isolates of *Escherichia coli*: ExPEC J Infect Dis 2000 181 5 1753 1754 10.1086/315418 10823778

[B3] Johnson JR Sannes MR Croy C Johnston B Clabots C Kuskowski MA Bender J Smith KE Winokur PL Belongia EA Antimicrobial drug–resistant *Escherichia coli* from humans and poultry products, Minnesota and Wisconsin, 2002–2004 Emerg Infect Dis 2007 13 6 838 10.3201/eid1306.061576 17553221 PMC2792839

[B4] Vincent C Boerlin P Daignault D Dozois CM Dutil L Galanakis C Reid-Smith RJ Tellier P-P Tellis PA Ziebell K Manges AR Food reservoir for *Escherichia coli* causing urinary tract infections Emerg Infect Dis 2010 16 1 88 10.3201/eid1601.091118 20031048 PMC2874376

[B5] Jakobsen L Garneau P Bruant G Harel J Olsen S Porsbo LJ Hammerum A Frimodt-Møller N Is *Escherichia coli* urinary tract infection a zoonosis? Proof of direct link with production animals and meat Eur J Clin Microbiol Infect Dis 2012 31 6 1121 1129 10.1007/s10096-011-1417-5 22033854

[B6] Gibreel TM Dodgson AR Cheesbrough J Fox AJ Bolton FJ Upton M Population structure, virulence potential and antibiotic susceptibility of uropathogenic *Escherichia coli* from Northwest England J Antimicrob Chemother 2012 67 2 346 356 10.1093/jac/dkr451 22028202

[B7] Kudinha T Johnson JR Andrew SD Kong F Anderson P Gilbert GL Escherichia coli sequence type 131 as a prominent cause of antibiotic resistance among urinary escherichia coli isolates from reproductive-age women J Clin Microbiol 2013 51 10 3270 3276 doi:10.1128/JCM.01315-13. [ http://jcm.asm.org/content/51/10/3270.full.pdf+html] 10.1128/JCM.01315-13 23885001 PMC3811657

[B8] Abraham S Wong HS Turnidge J Johnson JR Trott DJ Carbapenemase-producing bacteria in companion animals: a public health concern on the horizon J Antimicrob Chemother 2014 69 5 1155 1157 doi:10.1093/jac/dkt518. [ http://jac.oxfordjournals.org/content/69/5/1155.full.pdf+html] 10.1093/jac/dkt518 24398342

[B9] Platell JL Trott DJ Johnson JR Heisig P Heisig A Clabots CR Johnston B Cobbold RN Prominence of an O75 clonal group (clonal complex 14) among non-ST131 fluoroquinolone-resistant *Escherichia coli* causing extraintestinal infections in humans and dogs in Australia Antimicrob Agents Chemother 2012 56 7 3898 3904 10.1128/AAC.06120-11 22526317 PMC3393427

[B10] Tritt A Eisen JA Facciotti MT Darling AE An integrated pipeline for de novo assembly of microbial genomes PLoS ONE 2012 7 9 42304 doi:10.1371/journal.pone.0042304 10.1371/journal.pone.0042304 PMC3441570 23028432

[B11] Aziz R Bartels D Best A DeJongh M Disz T Edwards R Formsma K Gerdes S Glass E Kubal M Meyer F Olsen G Olson R Osterman A Overbeek R McNeil L Paarmann D Paczian T Parrello B Pusch G Reich C Stevens R Vassieva O Vonstein V Wilke A Zagnitko O The RAST Server: Rapid Annotations using Subsystems Technology BMC Genomics 2008 9 1 75 doi:10.1186/1471-2164-9-75 10.1186/1471-2164-9-75 18261238 PMC2265698

[B12] Darling AE Jospin G Lowe E Matsen IV FA Bik HM Eisen JA Phylosift: phylogenetic analysis of genomes and metagenomes PeerJ 2014 2 243 10.7717/peerj.243 PMC3897386 24482762

[B13] Price MN Dehal PS Arkin AP Fasttree 2–approximately maximum-likelihood trees for large alignments PloS one 2010 5 3 9490 10.1371/journal.pone.0009490 PMC2835736 20224823

[B14] Rissman AI Mau B Biehl BS Darling AE Glasner JD Perna NT Reordering contigs of draft genomes using the Mauve aligner Bioinformatics 2009 25 16 2071 2073 10.1093/bioinformatics/btp356 19515959 PMC2723005

[B15] Grant JR Stothard P The CGView Server: a comparative genomics tool for circular genomes Nucleic Acids Res 2008 36 suppl 2 181 184 10.1093/nar/gkn179 PMC2447734 18411202

