# Supplementary material for: A draft genome of Escherichia coli sequence type 127 strain 2009-46
Source: Gut Pathog. 2014 Sep 1;6:32. doi: 10.1186/1757-4749-6-32 (PMC4155142; doi:10.1186/1757-4749-6-32)
Supplement: Additional file 3 — Details on PCR cartography, virulence gene searches, and antibiotic resistance assays. [file 1757-4749-6-32-S3.pdf]

PCR cartography data

Isolate 2009-46 contained a novel 1500bp *intI1*-IS*26* configuration. It also returned no product for both cassette array and 3`-CS PCR screenings . This, in conjunction with positive results for *blaTEM-1, sul2, strA* and *strB*, lead us to believe that deletion of the cassette array and 3`-CS primer binding sites had occurred, with Tn*6029* implicated in the deletion (Table 1). This isolate represented the B2 phylotype.

Table 1:

**Gene or Element**	**2009-46**
intI1	+
intI2	-
IS26	+
aadA1	-
aadA5	-
dfrA5	-
dfrA7	-
dfrA17	-
sul2	+
strA	+
strB	+
blaTEM-1	+
merA	+
aphA1	-
Tn21	+
tetA	-

Virulence and resistance genes identified in the genome

Virulence genes:

**Category**	**Gene/Protein**	**2009-46**
**Tolerance to Colicin E2**	creA	+
	creB	+
	creC	+
	creD	+
**Colicin V & Bacteriocin Production Cluster**	R1	+
	dedA	+
	R3	+
	R4	+
	R5	+
	dedD	+
	Toxin	+

	purF	+
**MdtABCD Multidrug Resistance Cluster**	baeS	+
	baeR	+
	mdtA	
	mdtB	+
	mdtC	+
	mdtD	+
**Multidrug Resistance tripartite systems**	MFP	+
	IM	+
	OM	+
**Multiple Antibiotic Resistance Locus**	marR	+
	marA	+
	marB	+
	marC	+

Resistance genes :

**Category**	**Gene/Protein**	**2009-46**
**Resistance to Fluoroquinolones**	parC	+
	pare	+
	gyrA	+
	gyrB	+
	Lde	
**B-Lactamases**	BL	+
	BLc	
	BLI	+
**Multidrug Resistance Efflux Pumps**	cmeA	+
	cmeB	+
	TolC	+
	Reg	+
	MATE	+
	MFS	+
	macA	+
	macB	+
	OML	+
	RND	+

Sequence Typing

The genome sequence of the isolated was also uploaded onto the PubMLST to find the sequence type of the isolate. Result= ST127 by Actman's MLST scheme

CDS testing data

Antibiotic susceptibility of the isolate was assessed using the standard Calibrated Dichotomous Susceptibility (CDS) testing methodology outlined in the 2013 edition of the CDS manual (Bell et al 1999) and using Sensitest Agar (Oxoid®). Six different antibiotic discs (Oxoid®) were tested per 90mm Agar plate and dispensed using a 6-Cartridge Disc Dispenser (Oxoid®). The experiment included three reference strains (*E. coli* ACM5185, β-lactamase positive *E. coli* ACM5186 and *Pseudomonas aeruginosa* ACM5189) for quality control purposes.

**Antibiotic**$^{(\mu g)}$	**Annular Radii of inhibition zone (mm)**				***E.coli*** **2009-46 phenotype**
	**ACM 5185 (*E. coli*)**	**ACM 5186 (*E. coli*)**	**ACM 5189 (*P.aeruginosa*)**	***E.coli*** **2009-46**	
Augmentin$^{(60)}$	10	7	0	7	**S**
Cefotaxime$^{(5)}$	12	13	2	9	**S**
Ampicillin$^{(25)}$	11	0	0	0	**R**
Trimethoprim$^{(5)}$	12	12	0	0	**R**
Sulphafurazole$^{(300)}$	10	0	0	0	**R**
Ciprofloxacin$^{(2.5)}$	14	11	10	9	**S**
Cefoxitin$^{(30)}$	10	8	0	7	**S**
Timentin$^{(85)}$	9	5	7	6	**S**
Chloramphenicol$^{(30)}$	10	0	0	6.5	**S**
Cephalexin$^{(100)}$	7	7	0	7	**S**
Tetracycline$^{(10)}$*	6	0	2	0	**R**
Neomycin$^{(30)}$*	6	5	2	4.5	**S**
Streptomycin$^{(25)}$	6	1.5	5	0	**R**
Gentamicin$^{(10)}$*	7	6	7	5	**S**
Apramycin$^{(15)}$*	4	2.5	3	2	**R**
Imipenem$^{(10)}$	10	7	8	8	**S**

Kanamycin(50)	6	4	0	5	**R**
Azithromycin(15)*	5	4	0	3	**R**
Nalidixic Acid(30)	10	10	1	7.5	**S**

An asterisk indicates the isolate is being reported as susceptible for annular radii >4mm as opposed to >6mm. Antibiotics in italics are used in veterinary practice. All inhibitory zones in the 3 reference strains were within the recommended range (i.e., known susceptibilities for quality control).

References

Bell, S. M., B. J. Gatus, J. N. Pham, and D. L. Rafferty. "Antibiotic susceptibility testing by the CDS method." A concise laboratory manual. Arthur Productions Pty Ltd, Sydney. NSW: The antibiotic reference laboratory, South Eastern Area Laboratory Services (1999).
